# The Metabolically Active Bacterial Microbiome of Tonsils and Mandibular Lymph Nodes of Slaughter Pigs

**DOI:** 10.3389/fmicb.2015.01362

**Published:** 2015-12-15

**Authors:** Evelyne Mann, Beate Pinior, Stefanie U. Wetzels, Barbara U. Metzler-Zebeli, Martin Wagner, Stephan Schmitz-Esser

**Affiliations:** ^1^Institute of Milk Hygiene, Milk Technology and Food Science, Department for Farm Animals and Veterinary Public Health, University of Veterinary MedicineVienna, Austria; ^2^Research Cluster ‘Animal Gut Health’, Department for Farm Animals and Veterinary Public Health, University of Veterinary MedicineVienna, Austria; ^3^Institute for Veterinary Public Health, Department for Farm Animals and Veterinary Public Health, University of Veterinary MedicineVienna, Austria; ^4^Institute of Animal Nutrition and Functional Plant Compounds, Department for Farm Animals and Veterinary Public Health, University of Veterinary MedicineVienna, Austria; ^5^University Clinic for Swine, Department for Farm Animals and Veterinary Public Health, University of Veterinary MedicineVienna, Austria

**Keywords:** 16S rRNA sequencing, lymph node, tonsil, contamination, slaughter house, microbiota

## Abstract

The exploration of microbiomes in lymphatic organs is relevant for basic and applied research into explaining microbial translocation processes and understanding cross-contamination during slaughter. This study aimed to investigate whether metabolically active bacteria (MAB) could be detected within tonsils and mandibular lymph nodes (MLNs) of pigs. The hypervariable V1-V2 region of the bacterial 16S rRNA genes was amplified from cDNA from tonsils and MLNs of eight clinically healthy slaughter pigs. Pyrosequencing yielded 82,857 quality-controlled sequences, clustering into 576 operational taxonomic units (OTUs), which were assigned to 230 genera and 16 phyla. The actual number of detected OTUs per sample varied highly (23–171 OTUs). *Prevotella zoogleoformans* and *Serratia proteamaculans* (best type strain hits) were most abundant (10.6 and 41.8%, respectively) in tonsils and MLNs, respectively. To explore bacterial correlation patterns between samples of each tissue, pairwise Spearman correlations (*r*_s_) were calculated. In total, 194 strong positive and negative correlations |*r*_s_| ≥ 0.6 were found. We conclude that (i) lymphatic organs harbor a high diversity of MAB, (ii) the occurrence of viable bacteria in lymph nodes is not restricted to pathological processes and (iii) lymphatic tissues may serve as a contamination source in pig slaughterhouses. This study confirms the necessity of the EFSA regulation with regard to a meat inspection based on visual examinations to foster a minimization of microbial contamination.

## Introduction

Bacterial cross-contamination during pig slaughter is a hidden, invisible process, and is complex to monitor. The porcine gastrointestinal tract and its appending organs are considered most hazardous for microbial contamination of pork. The hazard deriving from lymphatic organs is comparatively low if tissues are not mobilized or opened up. However, a contamination during the handling of lymph nodes or tonsils while processing carcass halves is easily overlooked, particularly if lymphatic tissues lack any signs of pathological alteration. Pursuant to a former regulation of *post-mortem* pork inspection, Council Directive (EC) No. 854/2004, mandibular lymph nodes (MLNs) were required to be incised and examined during meat inspection (MI; European Parliament and the Council of the European Union, 2004). This directive has recently been repealed by a commission regulation with regard to an only visual MI. Additional palpation and incision of tissues, for example lymph nodes, is permitted if a possible risk to public health is suspected (European Commission, 2014). The regulation is intended to aim for an MI focusing on visual detection of pathologies and, consequently, minimalizing the probable microbial contamination which may derive from incised organs during MI.

This study was designed to provide valid information about the viable part of the bacterial diversity in lymphatic organs and to support this decision. The key question of this study was whether metabolically active bacteria (MAB) can be detected within healthy tonsils and MLNs of slaughter pigs.

Culture-independent methods have arisen during the last 20 years to assess the full diversity of microorganisms on food and to overcome cultivation biases (Giraffa and Neviani, 2001; Cocolin et al., 2011). High-throughput sequencing has revolutionized the ability to investigate bacterial microbiome compositions in farm animals and recently the diversity of microbes occupying different niches in the gastrointestinal tract, the soft palate and enteric lymph nodes of pigs has been described (Lowe et al., 2012; Mann et al., 2014a,b; Zhao et al., 2015). However, these methods have rarely been used for spoilage analysis of carcasses until now.

Palatine tonsils are often incompletely removed during MI. During the pig’s life, palatine tonsils are constantly involved in immune surveillance, whereby the crypts paradoxically provide a colonization niche for many commensal as well as pathogenic organisms (Horter et al., 2003). These organisms were primarily ingested or inhaled and therefore tonsils are also a routine sampling site for pathogen surveillance (Horter et al., 2003). Two culture-dependent and independent DNA-based community analyses of porcine tonsils (Lowe et al., 2011, 2012) and one study examining the impact of the porcine nasal microbiome on methicillin-resistant *Staphylococcus aureus* carriage (Weese et al., 2014) have been published recently, describing highly diverse microbial DNA patterns.

The MLN drains several superficial structures of the face, the intermandibular space and the nostrils area. A variety of invasive bacteria which translocate to the MLN in the mammalian body have been described so far, e.g., *Salmonella* Typhimurium or *Streptococcus suis* in pigs (Madsen et al., 2002; Vieira-Pinto et al., 2005), *Brucella microti* in red foxes (Scholz et al., 2009) or *Mycobacterium bovis* in boars (Naranjo et al., 2006). In an early study it was suggested that autochthonous bacteria are constantly translocating in low numbers from the gastrointestinal tract of mammals to the mesenteric lymph nodes (Berg, 1995). Bacteria were detected in mesenteric lymph nodes of healthy mice, transported by dendritic cells (Macpherson and Uhr, 2004; Macpherson and Smith, 2006; Obata et al., 2010). Recently, three independent in-depth sequencing studies have provided evidence that the occurrence of high loads of microbial DNA in lymph nodes is not restricted to invasive pathogens and that a diverse microbiome exists in the lymph nodes of pigs, rats, and mule deer (Wittekindt et al., 2010; Cuenca et al., 2014; Mann et al., 2014a). Even if cultivation efforts have proven the existence of viable bacteria in lymph nodes; e.g., bacilli, *Enterobacteriaceae, Clostridiaceae, Corynebacteriaceae*, and *Mycobacteriaceae* (Dahlinger et al., 1997; Pate et al., 2004; Mann et al., 2015), the diversity of the metabolically active part has not been investigated until now.

## Materials and Methods

### Sampling Procedure

Porcine tissues were sampled at an IFS (International Food Standard) certified slaughterhouse in Austria. The MI was carried out by veterinarians and included incision of the MLN and removal of the palatine tonsils. Carcass halves (*n* = 8) were sampled for residual tonsil tissue (1–2 cm^3^), which remained in the carcass after the initial removal of the tonsils, and the incised MLN. All pigs included in the study originate from different farms. The surface of the incised MLNs was disinfected by dipping the MLN into 70% ethanol. Subsequently, a half of the incised MLN was sampled: Biopsies of 1 g were taken from an uncut part of the MLN. Tonsil residues and MLN biopsies were cut into small pieces using sterile scalpel blades and all lymphatic tissues were stored separately in RNAlater (Life Technologies, Vienna, Austria) for 2–3 h at room temperature until RNA extraction.

### Extraction Procedures and Preparation of 16S rRNA Amplicon Libraries

RNA was isolated from 1 g tonsil and lymph node tissue using the Power Soil^TM^ Total RNA Isolation kit (Mo Bio Laboratories, Carlsbad, CA, USA) according to the manufacturer’s instructions. DNA was removed by the DNase Kit ‘Turbo DNA-free’ (Thermo Fisher Scientific, Vienna, Austria) and the integrity of the RNA was checked with an Agilent Bioanalyzer (Agilent Technologies, Palo Alto, CA, USA). RNA was transcribed into cDNA by the RevertAid H Minus First Strand cDNA Synthesis Kit (Thermo Fisher Scientific, Vienna, Austria) and adjusted to 25 ng/μl in DEPC-treated water (Fermentas GmbH, St. Leonia, NJ, USA). The V1–V2 region of 16S rRNA genes was amplified with the primers F27 (5′-AGA GTT TGA TCC TGG CTC AG-3′; Weisburg et al., 1991) and R357 (5′-CTG CTG CCT YCC GTA-3′; Dorsch and Stackebrandt, 1992). The PCR reactions (50 μl) included Fast Start Buffer (1x concentration), 2.5 U High Fidelity Enzyme, 200 μM each of dNTPs, 0.4 μM barcoded primers (Eurofins MWG, Ebersberg, Germany), 2.5 mM MgCl_2_, PCR-grade water (Roche Diagnostics, Mannheim, Germany) and 125 ng cDNA. After denaturation at 95°C (3 min), amplification was carried out by 38 cycles at 95°C (45 s), annealing at 56°C (45 s), and by extension at 72°C (1 min), with a final extension for 7 min. Amplicons were purified (Transgenomic Inc., Omaha, NE, USA). Subsequently, amplicons were eluted and DNA concentrations were determined with a PicoGreen^®^ dsDNA Assay Kit (Life Technologies, Carlsbad, CA, USA). Amplicons were quality checked using a 2100 Bio Analyzer (Agilent Technologies, Waldbronn, Germany). Emulsion PCRs of pooled samples were performed with the GS Titanium MV emulsion PCR Kit (Roche 454 Life Science).

### Pyrosequencing, Read Processing, and Statistics

Sequencing was performed with the GS-FLX Titanium Sequencing Kit “XLR70” (Roche 454 Life Science) at the Medical University of Graz (Center for Medical Research, Core Facility Molecular Biology, Austria). All sequences derived from GS-FLX sequencing were processed together with the software mothur, version 1.34.0 (Schloss et al., 2009). The description of the workflow, published elsewhere (Schloss and Westcott, 2011), served as template for processing. Low-quality sequences, primers and barcodes were trimmed with a minimum average quality score of 35 (window size of 50 bp), a minimum sequence length of 162 bp and an allowed number of differences to primer- and barcode- sequences of 2 and 1, respectively. A filter for maximal homopolymer length of 8 was applied. “pre.cluster” and “chimera.uchime” commands, both implemented in mothur, reduced sequencing errors and excluded chimeric sequences.

In total, 82,857 sequences passed the quality control. Uncorrected pairwise distances (“dist.seqs” command in mothur) were used as input for the assignment to OTUs (operational taxonomic units), which were calculated based on a distance limit of 0.03. In a second control step, OTUs containing less than five sequences were removed. The RDP naïve Bayesian rRNA Classifier (Wang et al., 2007) and the SILVA SSU reference database v102 (Pruesse et al., 2007) were used for taxonomic classification of sequences. The “classify.otu” command in mothur was used to assign a taxonomy to each OTU. For estimation of species diversity, data were bootstrapped (500 replicates) and species richness estimators and diversity indices were calculated with Explicet (Robertson et al., 2013).

Statistically significant differences in relative abundance with regard to sampling sites (MLNs and tonsils) were calculated using ‘metastats’ in mothur, which is based on the homonymous bioinformatics program (White et al., 2009). Means were reported ±standard deviation (SD). Fold-changes describe the abundance change of a phylotype of one sampling site compared to another sampling site. The significance level was set to *p* < 0.05. To control the false discovery rate at 10%, only significant phylotypes (*p* < 0.05) with *q*-values ≤ 0.1 were considered. The 30 most abundant OTUs of both sampling sites were additionally classified against type strains using the Greengenes database^1^ (DeSantis et al., 2006). The tonsil dataset was additionally compared with a published tonsil dataset, which was based on cloning and Sanger sequencing (Lowe et al., 2011). Sequences were processed together with mothur and a Venn diagram of OTUs (distance limit of 0.03) was generated with the Venn diagram plotter (PNNL, Richland, WA). Discriminant analyses were calculated in JMP Pro (SAS Institute, North Carolina, USA). The heatmap was created using JColorGrid (Joachimiak et al., 2006).

### Correlation Networks

In a next step, we focused on the mathematical and topological features of the pairwise Spearman correlations (*r*_s_) between the 30 most abundant genera within the eight samples of each tissue. In this context, *r*_s_ were computed for the MLNs and tonsils group. The resulting correlation matrix was converted into a network. According to graph theory, a graph or network (*G = V,E)* consists of nodes *V* (e.g., *v*_i_,*v*_j_) and edges *E* (Pinior et al., 2012a, 2015), where the nodes correspond to genera and the edges represent the correlation values between these genera [i.e., *e*_i_ *=* cor(*v*_i_,*v*_j_)]. Thus, an *e*_i_ can be considered as the pair of nodes it connects (Pinior et al., 2012b), whereby the relation between the nodes can be weighted (e.g., correlation values). In the next step, the graph was visualized as an arc-diagram^2^. The network calculations and visualizations were implemented using the R statistical computing environment (R Core Team, 2014^3^).

### Accession Number of Pyrosequencing Data

Pyrosequencing data are available in the EMBL SRA database under the accession number PRJEB9066.

## Results

All reads derived from pyrosequencing (16 samples) were processed together. Between 2,779 and 8,952 pyrotags per sample passed the quality control. A total of 576 OTUs were assigned containing more than five sequences per OTU and these OTUs were used for all downstream analyses. Species richness and diversity indices for both sampling sites are listed in **Table 1**. Values of richness and diversity indices did not differ significantly among sampling sites, but species richness estimators indicate a slightly higher bacterial diversity in tonsils compared to MLNs.

**Table 1:** Species richness and diversity measures of tonsils and mandibular lymph nodes (MLNs). Mean values ± standard deviation are listed.

Sampling site	No. of species observed	Species richness		Diversity measures	
		ACE^1^	Chao 1	Shannon H	Simpson
Tonsils	97.25 ± 27.32	120.33 ± 36.32	121.75 ± 40.61	4.44 ± 0.40	0.09 ± 0.02
Mandibular lymph nodes	67.92 ± 43.86	77.72 ± 52.32	77.65 ± 52.76	3.19 ± 1.13	0.26 ± 0.14

*ACE^1^, abundance-based coverage estimator.*

Rarefaction curves were calculated for all samples and were depicted in Supplementary Figure S1A. High diversity coverage was achieved for MLNs with curves reaching asymptotes. Tonsils samples were sequenced sufficiently, but some tonsil curves did not reach complete saturation.

The actual number of detected OTUs varied highly among samples (23–171 OTUs). MLNs harbored between 23 and 171 OTUs (median = 66 OTUs) and tonsils between 49 and 158 OTUs (median = 105 OTUs). The total microbial diversity detected in MLNs and tonsils consisted of 355 OTUs and 278 OTUs, respectively.

**FIGURE 1:**
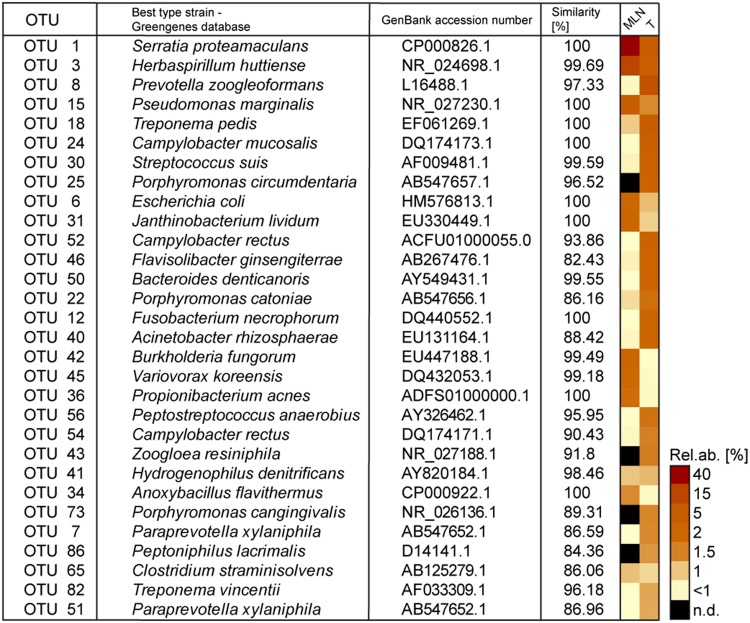
**The 30 most abundant operational taxonomic units (OTUs) detected in tonsils and mandibular lymph nodes (MLNs) are shown in the heatmap.** The best type strain hits (Greengenes database) and its accession numbers as well as similarity percentage is listed. Sampling sites were abbreviated as follows: T, tonsils; MLNs, mandibular lymph nodes. N.d. = not detected.

Rank abundance curve dynamics (Supplementary Figure S1B) were similar to dynamics found in complex microbial communities of other biological niches (e.g., gastrointestinal microbiomes). A high proportion of very low abundant OTUs was dominated by some highly abundant OTUs: 10 OTUs of MLNs and 23 OTUs of tonsils reached relative abundances of >1%. The 30 most abundant OTUs over both sampling sites are depicted in **Figure 1**. Detailed information on relative abundances of OTUs and the associated *p*-values are listed in Supplementary Table S1. In tonsils, OTU 8, OTU 18, and OTU 1 dominated (best hits: *Prevotella zoogleoformans, Treponema pedis*, and *Serratia proteamaculans*) with relative abundances between 5.3 and 10.6%. In MLNs, OTU 1 (best hit: *Serratia proteamaculans*) was highly dominant (41.8% of all sequences), followed by OTU 15 and OTU 3 (best hits: *Pseudomonas marginalis* and *Herbaspirillum huttiense*) with 5.6 and 4.1% relative abundance (**Figure 1**). All closest reference strains of the OTUs detected in our study have previously been described as occurring in the gastrointestinal tract of mammals, on plants or environmental samples.

**FIGURE 2:**
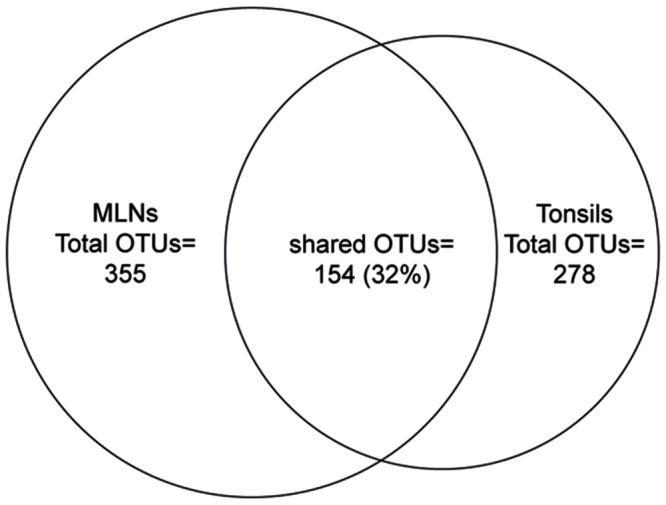
**Venn diagram showing the number of shared OTUs between the two sampling sites.** The size of the circles is in proportion to the number of OTUs detected in each sampling site.

Considering all 576 OTUs, 32% were shared between tonsil and MLN samples (**Figure 2**). These shared OTUs include also highly abundant OTUs: From the 30 most abundant OTUs, 87% were detected in both sampling sites. Comparing the relative abundances of OTUs between the two datasets, OTU 8 (best hit: *Prevotella zoogleoformans*), OTU 24 (best hit: *Campylobacter mucosalis*), OTU 12 (best hit: *Fusobacterium necrophorum*), and OTU 56 (best hit: *Peptostreptococcus anaerobius*) were significantly increased in tonsils compared to MLN tissues (106.0-, 88.6-, 510.0-, and 91.4-fold change, respectively). OTU 45 (best hit: *Variovorax koreensis*) was significantly enriched in MLNs compared to tonsils (26.8-fold change).

**FIGURE 3:**
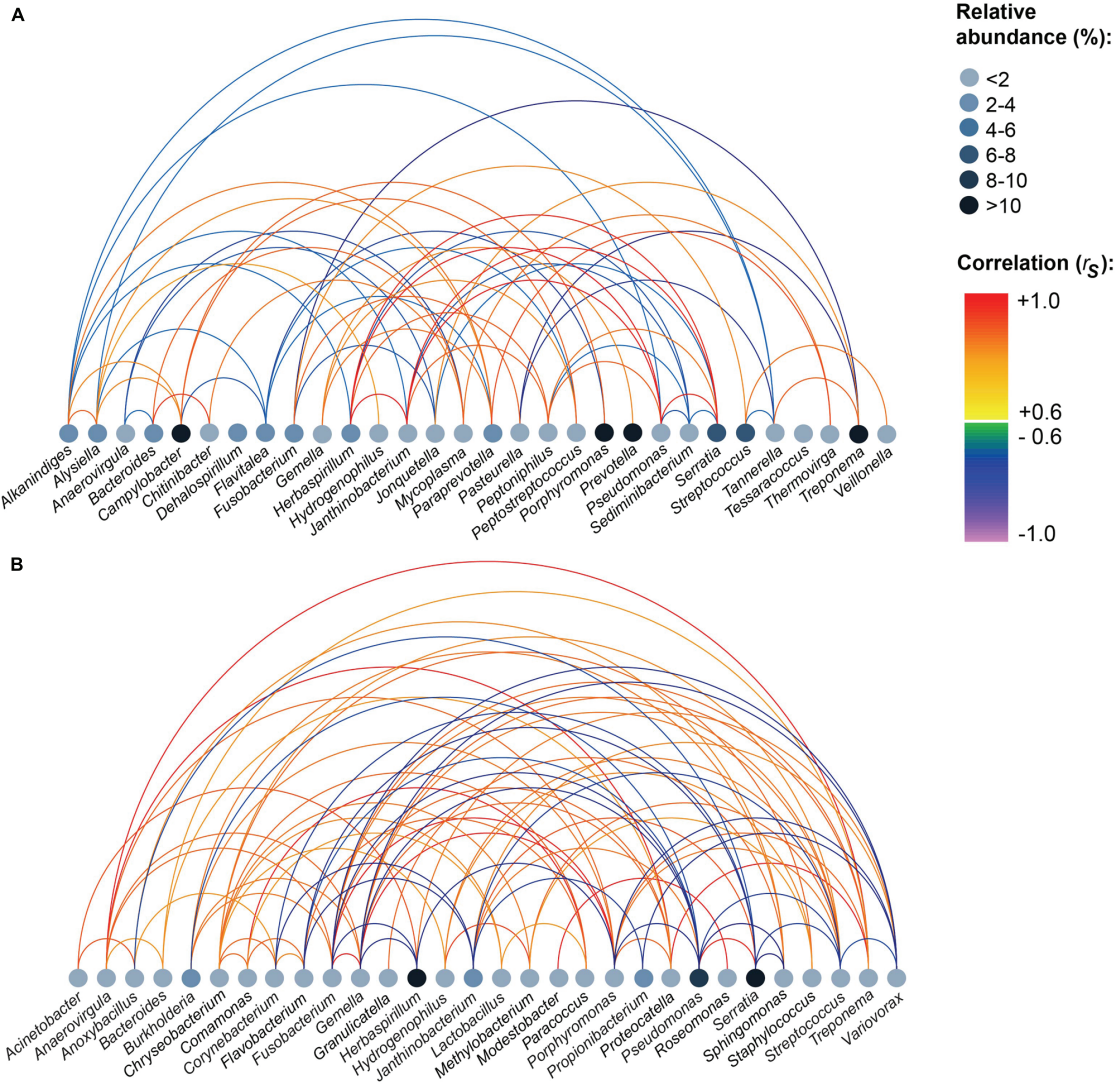
**Strong positive and negative correlations between genera within all samples of each sampling site. (A)** Tonsils, **(B)** mandibular lymph nodes. Pairwise Spearman correlations (*r_s_*) between the most abundant genera were computed for each tissue. Relative abundances of genera are indicated by node color. The color of the edges indicates the degree of correlation. The figure depicts correlations |*r_s_*| ≥ 0.6.

Based on the mothur OTU classification, we assigned all OTUs to their respective genus (*n* = 230). The complete list of genera detected in each tissue and relative abundances of genera and *p*-values can be found in Supplementary Tables S2 and S3. In accordance to results on OTU level, *Serratia, Herbaspirillum*, and *Pseudomonas* were highly abundant in MLNs, whereas *Prevotella, Treponema, Campylobacter*, and *Porphyromonas* were highly abundant in tonsils. To explore bacterial correlation similarities between samples in a tissue group, pairwise Spearman correlations (*r*_s_) were calculated for abundant genera. **Figure 3** shows that in the tonsil network *Pseudomonas, Herbaspirillum, Serratia*, and *Janthinobacterium* highly positively correlated among each other (*r*_s_ *>* 0.9). A strong negative correlation (*r*_s_ *=* –0.9) was calculated for *Treponema* and *Pasteurella*. In the MLN network *Treponema, Anaerovirgula*, and *Proteocatella* highly positively correlated with each other (*r*_s_ *>* 0.9). Highly positive correlations were also found between *Gemella, Porphyromonas*, and *Fusobacterium* (*r*_s_ *>* 0.9). Additionally, *Comamonas* and *Paracoccus, Porphyromonas* and *Fusobacterium, Modestobacter* and *Roseomonas*, and *Serratia* and *Pseudomonas* highly positively correlated (*r*_s_ *>* 0.9).

**FIGURE 4:**
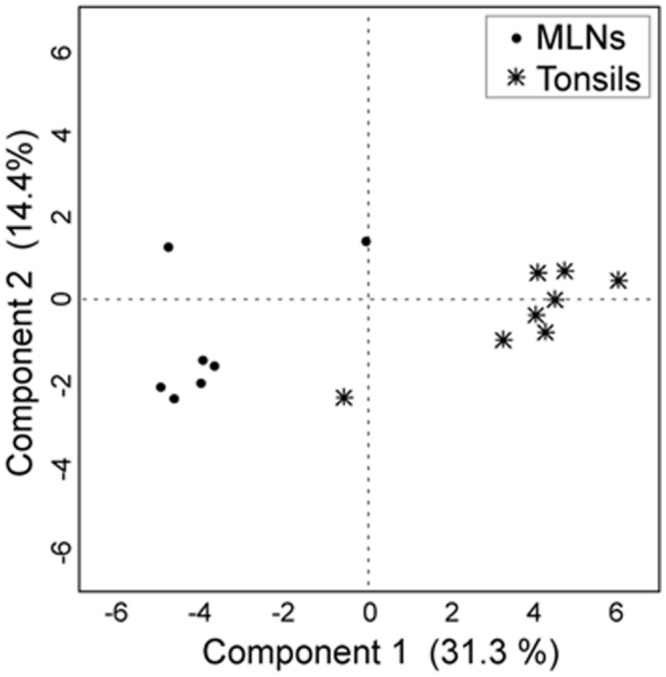
**Discriminant analysis with the first two principal components.** For the discriminant analysis the 30 most abundant genera were used as covariates and the sampling site as the categorial variable. Each point corresponds to one sample.

Tonsils and MLN samples clustered distinctly in the discriminant analysis (**Figure 4**). In the MLN dataset, *Serratia, Herbaspirillum, Pseudomonas, Janthinobacterium*, and *Variovorax* had the highest component loadings. In the tonsil dataset, *Prevotella, Paraprevotella, Porphyromonas*, and *Peptostreptococcus* vectors had highest loadings (loading plot data not shown).

**FIGURE 5:**
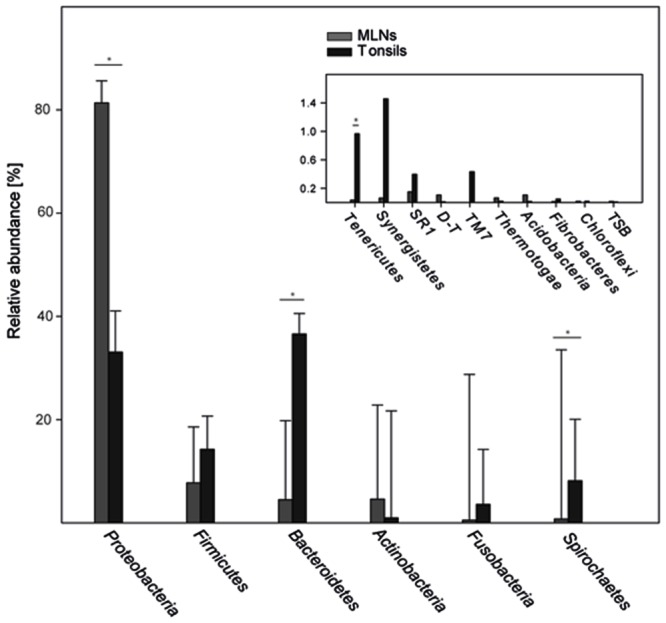
**Relative abundances of bacterial phyla.** Abundant phyla with more than 2% mean abundance are shown for each sampling site (tonsils, MLNs = mandibular lymph nodes). In the inlay, relative abundances of rare phyla (<2% mean abundance) are depicted. The phylum *Deinococcus–Thermus* is abbreviated with *D–T*, Thermodesulfobacteria with *TSB*. Error bars represent standard error of the means. Significance in one sampling site compared to another is marked with an asterisk and is defined as *p* ≤ 0.05 and *q* ≤ 0.1.

In total 16 phyla were identified, *Proteobacteria, Firmicutes*, and *Bacteroidetes* being most abundant (86.6% of all reads; **Figure 5**). *Proteobacteria* was significantly lower in tonsils compared to MLNs (2.5-fold change). *Bacteroidetes, Spirochaetes*, and *Tenericutes* were significantly increased in tonsils compared to MLNs (8.1-, 11.7-, and 28.4-fold change, respectively). Exact values of relative abundances at phylum level, SD and *p*-values are available in Supplementary Table S4.

## Discussion

In this study, the metabolically active part of the microbiome of cervical lymphatic tissues of slaughter pigs was examined for the first time.

Being aware of current discussions about artifacts that might be introduced by pyrosequencing (Quince et al., 2009, 2011), we set up stringent quality controls to reduce potential sequencing biases. However, reads from pyrosequencing are short (median value = 182 bp) and therefore the ‘best hits’ provided in this manuscript should be interpreted carefully, especially if sequence similarity is below 99%.

### The Metabolically Active Microbiome of Porcine Palatine Tonsils

Pigs have five types of tonsils including lingual, paraepiglottic, pharyngeal, tubal, and palatine tonsils. Tonsils contain tonsillar crypts and lymphoid follicles with M cells located within deep crypts, aiming at providing a first ‘handling site’ for host immune cells (Horter et al., 2003). Although tonsils are routinely used in porcine disease surveillance for many pathogens (Bonardi et al., 2003; Wertheim et al., 2009), the nasal and palatine region of pigs has previously only been investigated to a limited extent concerning the diversity of microorganisms. In this study, we describe a substantial MAB diversity in palate tonsils. In contrast to our RNA-based approach, Lowe et al. used a DNA-based approach for examining the tonsil microbiome (Lowe et al., 2012). The actual richness detected in tonsils in the DNA-based approach amounted to four times the number of OTUs found in our study. This can be explained by a high number of dead bacteria that had already been processed by the host immune cells or bacterial DNA fragments detected. The high number of OTU 8 (best hit: *Prevotella zoogleoformans*) and OTU 18 (best hit: *Treponema pedis*) in tonsil samples was not surprising: In a study examining the porcine nasal microbiota based on DNA-based sequencing, *Treponema* had already been described as being the predominant microorganism (Weese et al., 2014). *Prevotella* are well-known colonizers of human tonsils as well (Jensen et al., 2013). The genus *Prevotella* is also known to be an abundant colonizer of the pig’s gastrointestinal tract, especially of the colonic region (46% relative abundance) and *Prevotella* consists of a huge variety of bacterial species (Mann et al., 2014b). The correlation network of tonsils revealed strong correlations between *Pseudomonas, Herbaspirillum, Serratia*, and *Janthinobacterium* indicating a constant co-occurrence pattern of these genera in all tonsil samples. *Janthinobacterium* and *Pseudomonas* were low abundant species which might benefit from the presence of *Serratia*, which can break down many carbon sources.

A previous study based on DNA-sequencing defined a core microbiome (*Actinobacillus, Alkanindiges, Fusobacterium, Haemophilus, Lactobacillus, Moraxella, Pasteurella, Peptostreptococcus, Streptococcus*, and *Veillonella*) of pig tonsils based on the examination of two healthy pig herds (Lowe et al., 2012). With the exception of *Haemophilus*, all genera listed were also detected in our study, confirming the results of the above-mentioned study but also showing that these organisms are viable in tonsils.

In accordance with the results reported here, we suggest that *Prevotella, Paraprevotella, Porphyromonas, Treponema*, and *Campylobacter* should be included in the definition of the core microbiome of porcine palatine tonsils, as these four organisms were highly abundant in our MAB tonsil dataset (between 5% and 18% relative abundance) and were present in all tonsils examined. These organisms, together with the ‘core genera’ defined by Lowe et al. (2012) accounted for 51.3% of all sequences. The organisms suggested above were also detected in Lowe’s DNA-based sequencing study (Lowe et al., 2012), but in lower abundance. At phylum level, the DNA-based sequencing study of Lowe et al. (2012) described 73% *Proteobacteria* in tonsils. Interestingly, *Bacteroidetes*, which was the most abundant metabolically active phylum in tonsils in our study, were described as low abundant (0.8% of total sequences) by Lowe et al. (2012). One explanation would be a high proportion of dead *Proteobacteria* that may have overwhelmed metabolically active *Bacteroidetes* and other phyla in DNA-based datasets. This would be in accordance with cultivation data of microbes from porcine tonsils, where only about 20% could be assigned to genera belonging to *Proteobacteria* (Lowe et al., 2011). However, as stated by Lowe et al. (2012), a possible amplification bias during PCR reactions in the DNA-based approach would also explain this difference. Interestingly, a DNA-based deep sequencing study examining human tonsils described a similar proportion of phyla in healthy adults as detected in our study, and found a high proportion of *Proteobacteria* linked with recurrent tonsillitis in children (Jensen et al., 2013). Although we assume that the *Bacteroidetes* fraction might have been strongly underestimated by Lowe et al. (2011), conclusions about a possible pathological process linked to a high number of *Proteobacteria* would need further investigations. In another study, Lowe et al. (2011) provided extensive DNA-based 16S rRNA clone libraries from pig tonsils. We processed these sequences together with our tonsil dataset and revealed 30 OTUs shared, which cover 47.3% of all sequences. This indicates a substantial overlap of abundant OTUs (Supplementary Figure S2).

### The Metabolically Active Microbiome of Porcine MLN

In this study, we provide the first metabolically active microbiome of porcine MLNs consisting of commensals and pathogens. Thus, translocation of bacteria from the area of distribution to local lymph nodes is not restricted to pathological processes. The samples harbored between 23 and 171 OTUs (median = 66 OTUs), being in accordance with the number of OTUs detected in a DNA-based in-depth sequencing study of ileocaecal lymph nodes (Mann et al., 2014a). Previous studies have shown that the number of OTUs can vary widely in healthy (but also in pathologically altered) ileocaecal lymph nodes (Mann et al., 2014a). We assume that the exact sampling location (T-cell area fully covered or not) could contribute to differences in diversity. The overall diversity of MAB found in MLN was unexpectedly high (390 OTUs) and accounted for 60% of OTUs detected in ileocaecal lymph nodes with a DNA-based survey (Mann et al., 2014a). Interestingly 48.7% of all MLN sequences accounted for *Serratia*, of which 86% belonged to OTU 1 (best hit: *Serratia proteamaculans*). All pigs examined in this study harbored a high abundance of *Serratia proteamaculans* in MLNs. In the early 1950s, this Gram-negative, facultatively anaerobic bacterium was already proven to survive in lymph node tissue (Lepovetsky et al., 1953). *Serratia proteamaculans* was detected in moderate abundance (0.5%) in ileocaecal lymph nodes with the DNA-based survey (Mann et al., 2014a). Considering the high abundance of metabolically active *Serratia* which was found in this study, we assume that physiological conditions in lymph nodes foster their survival. *Serratia proteamaculans* has previously been isolated from human pneumonia samples, animal habitats and other environmental samples, indicating the flexibility of the growth of this species (Bollet et al., 1993; Dworkin and Falkow, 2006; Kajikazawa et al., 2007). It is known to be able to utilize a huge variety of carbon sources (Bollet et al., 1993; Dworkin and Falkow, 2006), and to be an opportunistic food-borne pathogen (Kajikazawa et al., 2007).

*Herbaspirillum*, also detected in high abundance (19.1%) in all pigs in our study, was not found in ileocaecal lymph nodes (Mann et al., 2014a). In total, 83% of *Herbaspirillum* sequences belonged to OTU 3 (best hit: *Herbaspirillum huttiense*), commonly found in soil- and animal- environments but also known to be present in human respiratory tracts (Spilker et al., 2008). It has been suggested that *Herbaspirillum huttiense* may contribute to lung diseases in humans (Spilker et al., 2008). In the MLN network, the number of strong correlations was more than double than that of the tonsil network. To our knowledge, most of the correlating genera (e.g., *Porphyromonas* or *Treponema*) have not previously been described in porcine lymph nodes until now.

Present results indicate that translocation processes to local lymph nodes are not restricted to lymph nodes draining the gastrointestinal tract, and are not restricted to pathological processes. Interestingly, the relatively high number of MAB found in pigs in the present study give us a direct hint to a possible survival scenario of various species in host immune cells. Dendritic cells for example are known to keep living bacteria and are ineffective in eliminating them, in contrast to macrophages (Macpherson and Uhr, 2004; Macpherson and Smith, 2006). However, further research investigating the exact position and function of MAB in lymph nodes is required.

This is the first RNA-based study exploring MAB in porcine lymphatic organs. Further studies using mRNA-based approaches might give insights into gene expression of the MAB and hence contribute to an increased understanding of microbial activity in lymphatic organs. With the methodological toolbox used, the relatedness between the bacterial community in lymphatic organs, the gut bacterial community and the slaughterhouse-specific bacterial community could be characterized and compared to link the occurrence of phylotypes to pork contamination in ongoing studies.

## Conclusion

This study confirms the necessity of the EFSA regulation with regard to an MI based on visual MI and assures that this decision can contribute to minimalize microbial contamination.

## Conflict of Interest Statement

The authors declare that the research was conducted in the absence of any commercial or financial relationships that could be construed as a potential conflict of interest.

## Footnotes

1 http://greengenes.lbl.gov

2 http://gastonsanchez.com/software/arcdiagram_introduction.pdf

3 http://www.r-project.org

## References

[B1] Berg R. D. (1995). Bacterial translocation from the gastrointestinal tract. *Trends Microbiol.* 3 149–154. 10.1016/S0966-842X(00)88906-4 7613757

[B2] Bollet C. Grimont P. Gainnier M. Geissler A. Sainty J. M. Demicco P. (1993). Fatal pneumonia due to *Serratia proteamaculans* subsp. quinovora. *J. Clin. Microbiol.* 31 444–445. 8432835 10.1128/jcm.31.2.444-445.1993 PMC262785

[B3] Bonardi S. Brindani F. Pizzin G. Lucidi L. D’incau M. Liebana E. (2003). Detection of *Salmonella* spp., *Yersinia enterocolitica* and verocytotoxin-producing *Escherichia coli* O157 in pigs at slaughter in Italy. *Int. J. Food Microbiol.* 85 101–110. 10.1016/S0168-1605(02)00504-4 12810275

[B4] Cocolin L. Dolci P. Rantsiou K. (2011). Biodiversity and dynamics of meat fermentations: the contribution of molecular methods for a better comprehension of a complex ecosystem. *Meat Sci.* 89 296–302. 10.1016/j.meatsci.2011.04.011 21555189

[B5] Cuenca S. Sanchez E. Santiago A. El Khader I. Panda S. Vidal S. (2014). Microbiome composition by pyrosequencing in mesenteric lymph nodes of rats with CCl4-induced cirrhosis. *J. Innate Immun.* 6 263–271. 10.1159/000356454 24296725 PMC6741495

[B6] Dahlinger J. Marks S. L. Hirsh D. C. (1997). Prevalence and identity of translocating bacteria in healthy dogs. *J. Vet. Int. Med.* 11 319–322. 10.1111/j.1939-1676.1997.tb00473.x 9470154

[B7] DeSantis T. Z. Hugenholtz P. Larsen N. Rojas M. Brodie E. L. Keller K. (2006). Greengenes, a chimera-checked 16S rRNA gene database and workbench compatible with ARB. *Appl. Environ. Microbiol.* 72 5069–5072. 10.1128/AEM.03006-05 16820507 PMC1489311

[B8] Dorsch M. Stackebrandt E. (1992). Some modifications in the procedure of direct sequencing of PCR amplified 16S rDNA. *J. Microbiol. Methods* 16 271–279. 10.1016/0167-7012(92)90017-X

[B9] Dworkin M. Falkow S. (2006). *The Prokaryotes: Proteobacteria, Gamma Subclass*. New York, NY: Springer Science and Business Media1.

[B10] European Commission (2014). Annexes to regulation (EC) No 853/2004 and (EC) No 854/2004 of the european parliament and of the council and commission regulation (EC) No 2074/2005. *OJEUL* 69 95–98.

[B11] European Parliament and the Council of the European Union (2004). Regulation (EC) No 854/2004 of the european parliament and of the council of 29 April 2004 laying down specific rules for the organisation of official controls on products of animal origin intended for human consumption. *OJEUL* 226 83–127.

[B12] Giraffa G. Neviani E. (2001). DNA-based, culture-independent strategies for evaluating microbial communities in food-associated ecosystems. *Int. J. Food Microbiol.* 67 19–34. 10.1016/S0168-1605(01)00445-7 11482566

[B13] Horter D. C. Yoon K. J. Zimmerman J. J. (2003). A review of porcine tonsils in immunity and disease. *Anim. Health. Res. Rev.* 4 143–155. 10.1079/AHRR200358 15134296

[B14] Jensen A. Fago-Olsen H. Sorensen C. H. Kilian M. (2013). Molecular mapping to species level of the tonsillar crypt microbiota associated with health and recurrent tonsillitis. *PLoS ONE* 8:e56418 10.1371/journal.pone.0056418 PMC3578847 23437130

[B15] Joachimiak M. P. Weisman J. L. May B. (2006). ColorGrid: software for the visualization of biological measurements. *BMC Bioinformatics* 7:225 10.1186/1471-2105-7-225 PMC1479842 16640789

[B16] Kajikazawa T. Sugita T. Nishikawa A. (2007). Comprehensive identification of bacteria in processed fresh edible sea urchin using 16S ribosomal DNA sequence analysis: the products contain various food poisoning-related bacteria and opportunistic bacterial pathogens. *J. Health Sci.* 53 756–759. 10.1248/Jhs.53.756

[B17] Lepovetsky B. C. Weiser H. H. Deatherage F. E. (1953). A microbiological study of lymph nodes, bone marrow and muscle tissue obtained from slaughtered cattle. *Appl. Microbiol.* 1 57–59. 13008432 10.1128/am.1.1.57-59.1953 PMC1056859

[B18] Lowe B. A. Marsh T. L. Isaacs-Cosgrove N. Kirkwood R. N. Kiupel M. Mulks M. H. (2011). Microbial communities in the tonsils of healthy pigs. *Vet. Microbiol.* 147 346–357. 10.1016/j.vetmic.2010.06.025 20663617

[B19] Lowe B. A. Marsh T. L. Isaacs-Cosgrove N. Kirkwood R. N. Kiupel M. Mulks M. H. (2012). Defining the “core microbiome” of the microbial communities in the tonsils of healthy pigs. *BMC Microbiol.* 12:20 10.1186/1471-2180-12-20 PMC3297519 22313693

[B20] Macpherson A. J. Smith K. (2006). Mesenteric lymph nodes at the center of immune anatomy. *J. Exp. Med.* 203 497–500. 10.1084/Jem.20060227 16533891 PMC2118258

[B21] Macpherson A. J. Uhr T. (2004). Induction of protective IgA by intestinal dendritic cells carrying commensal bacteria. *Science* 303 1662–1665. 10.1126/science.1091334 15016999

[B22] Madsen L. W. Bak H. Nielsen B. Jensen H. E. Aalbaek B. Riising H. J. (2002). Bacterial colonization and invasion in pigs experimentally exposed to Streptococcus suis serotype 2 in aerosol. *J. Vet. Med. B Infect. Dis. Vet. Public Health* 49 211–215. 10.1046/j.1439-0450.2002.00491.x 12121040

[B23] Mann E. Dzieciol M. Metzler-Zebeli B. U. Wagner M. Schmitz-Esser S. (2014a). Microbiomes of unreactive and pathologically altered ileocecal lymph nodes of slaughter pigs. *Appl. Environ. Microbiol.* 80 193–203. 10.1128/AEM.03089-13 24141125 PMC3911030

[B24] Mann E. Schmitz-Esser S. Zebeli Q. Wagner M. Ritzmann M. Metzler-Zebeli B. U. (2014b). Mucosa-associated bacterial microbiome of the gastrointestinal tract of weaned pigs and dynamics linked to dietary calcium-phosphorus. *PLoS ONE* 9:e86950 10.1371/journal.pone.0086950 PMC3900689 24466298

[B25] Mann E. Dzieciol M. Pinior B. Neubauer V. Metzler-Zebeli B. U. Wagner M. (2015). High diversity of viable bacteria isolated from lymph nodes of slaughter pigs and its possible impacts for food safety. *J. Appl. Microbiol.* 119 1420–1432. 10.1111/jam.12933 26283649

[B26] Naranjo V. Hofle U. Vicente J. Martin M. P. Ruiz-Fons F. Gortazar C. (2006). Genes differentially expressed in oropharyngeal tonsils and mandibular lymph nodes of tuberculous and nontuberculous European wild boars naturally exposed to *Mycobacterium bovis*. *FEMS Immunol. Med. Microbiol.* 46 298–312. 10.1111/j.1574-695X.2005.00035.x 16487312

[B27] Obata T. Goto Y. Kunisawa J. Sato S. Sakamoto M. Setoyama H. (2010). Indigenous opportunistic bacteria inhabit mammalian gut-associated lymphoid tissues and share a mucosal antibody-mediated symbiosis. *Proc. Natl. Acad. Sci. U.S.A.* 107 7419–7424. 10.1073/pnas.1001061107 20360558 PMC2867693

[B28] Pate M. Pirs T. Zdovc I. Krt B. Ocepek M. (2004). Haemolytic *Rhodococcus equi* isolated from a swine lymph node with granulomatous lesions. *J. Vet. Med. B Infect. Dis. Vet. Public Health* 51 249–250. 10.1111/j.1439-0450.2004.00758.x 15330986

[B29] Pinior B. Conraths F. Petersen B. Selhorst T. (2015). Decision support for risk managers in the case of deliberate food contamination: the dairy industry as an example. *Omega* 53 41–48. 10.1016/j.omega.2014.09.011

[B30] Pinior B. Konschake M. Platz U. Thiele H. D. Petersen B. Conraths F. J. (2012a). The trade network in the dairy industry and its implication for the spread of contamination. *J. Dairy Sci.* 95 6351–6361. 10.3168/jds.2012-5809 22999280

[B31] Pinior B. Platz U. Ahrens U. Petersen B. Conraths F. Selhorst T. (2012b). The German milky way: trade structure of the milk industry, and possible consequences of a food crisis. *J. Chain. Netw. Sci.* 12 25–39. 10.3920/JCNS2012.x001

[B32] Pruesse E. Quast C. Knittel K. Fuchs B. M. Ludwig W. Peplies J. (2007). SILVA: a comprehensive online resource for quality checked and aligned ribosomal RNA sequence data compatible with ARB. *Nucleic Acids Res.* 35 7188–7196. 10.1093/nar/gkm864 17947321 PMC2175337

[B33] Quince C. Lanzen A. Curtis T. P. Davenport R. J. Hall N. Head I. M. (2009). Accurate determination of microbial diversity from 454 pyrosequencing data. *Nat. Methods* 6 639–641. 10.1038/nmeth.1361 19668203

[B34] Quince C. Lanzen A. Davenport R. J. Turnbaugh P. J. (2011). Removing noise from pyrosequenced amplicons. *BMC Bioinformatics* 12:38 10.1186/1471-2105-12-38 PMC3045300 21276213

[B35] R Core Team (2014). *R: A Language and Environment for Statistical Computing*. Vienna: R Foundation for Statistical Computing.

[B36] Robertson C. E. Harris J. K. Wagner B. D. Granger D. Browne K. Tatem B. (2013). Explicet: graphical user interface software for metadata-driven management, analysis and visualization of microbiome data. *Bioinformatics* 29 3100–3101. 10.1093/bioinformatics/btt526 24021386 PMC3834795

[B37] Schloss P. D. Westcott S. L. (2011). Assessing and improving methods used in operational taxonomic unit-based approaches for 16S rRNA gene sequence analysis. *Appl. Environ. Microbiol.* 77 3219–3226. 10.1128/AEM.02810-10 21421784 PMC3126452

[B38] Schloss P. D. Westcott S. L. Ryabin T. Hall J. R. Hartmann M. Hollister E. B. (2009). Introducing mothur: open-source, platform-independent, community-supported software for describing and comparing microbial communities. *Appl. Environ. Microbiol.* 75 7537–7541. 10.1128/AEM.01541-09 19801464 PMC2786419

[B39] Scholz H. C. Hofer E. Vergnaud G. Le Fleche P. Whatmore A. M. Al Dahouk S. (2009). Isolation of *Brucella microti* from mandibular lymph nodes of red foxes, Vulpes vulpes, in lower Austria. *Vector Borne Zoonotic Dis.* 9 153–156. 10.1089/vbz.2008.0036 18973444

[B40] Spilker T. Uluer A. Z. Marty F. M. Yeh W. W. Levison J. H. Vandamme P. (2008). Recovery of *Herbaspirillum* species from persons with cystic fibrosis. *J. Clin. Microbiol.* 46 2774–2777. 10.1128/JCM.00460-08 18524958 PMC2519502

[B41] Vieira-Pinto M. Temudo P. Martins C. (2005). Occurrence of *Salmonella* in the ileum, ileocolic lymph nodes, tonsils, mandibular lymph nodes and carcasses of pigs slaughtered for consumption. *J. Vet. Med. B Infect. Dis. Vet. Public Health* 52 476–481. 10.1111/j.1439-0450.2005.00892.x 16364024

[B42] Wang Q. Garrity G. M. Tiedje J. M. Cole J. R. (2007). Naive Bayesian classifier for rapid assignment of rRNA sequences into the new bacterial taxonomy. *Appl. Environ. Microbiol.* 73 5261–5267. 10.1128/AEM.00062-07 17586664 PMC1950982

[B43] Weese J. S. Slifierz M. Jalali M. Friendship R. (2014). Evaluation of the nasal microbiota in slaughter-age pigs and the impact on nasal methicillin-resistant *Staphylococcus aureus* (MRSA) carriage. *BMC Vet. Res.* 10:69 10.1186/1746-6148-10-69 PMC3995533 24628871

[B44] Weisburg W. G. Barns S. M. Pelletier D. A. Lane D. J. (1991). 16S ribosomal DNA amplification for phylogenetic study. *J. Bacteriol.* 173 697–703. 1987160 10.1128/jb.173.2.697-703.1991 PMC207061

[B45] Wertheim H. F. Nghia H. D. Taylor W. Schultsz C. (2009). *Streptococcus suis*: an emerging human pathogen. *Clin. Infect. Dis.* 48 617–625. 10.1086/596763 19191650

[B46] White J. R. Nagarajan N. Pop M. (2009). Statistical methods for detecting differentially abundant features in clinical metagenomic samples. *PLoS Comput. Biol.* 5:e1000352 10.1371/journal.pcbi.1000352 PMC2661018 19360128

[B47] Wittekindt N. E. Padhi A. Schuster S. C. Qi J. Zhao F. Tomsho L. P. (2010). Nodeomics: pathogen detection in vertebrate lymph nodes using meta-transcriptomics. *PLoS ONE* 5:e13432 10.1371/journal.pone.0013432 PMC2956653 20976145

[B48] Zhao W. Wang Y. Liu S. Huang J. Zhai Z. He C. (2015). The dynamic distribution of porcine microbiota across different ages and gastrointestinal tract segments. *PLoS ONE* 10:e0117441 10.1371/journal.pone.0117441 PMC4331431 25688558

