# Supplementary material for: The Metabolically Active Bacterial Microbiome of Tonsils and Mandibular Lymph Nodes of Slaughter Pigs
Source: Front Microbiol. 2015 Dec 15;6:1362. doi: 10.3389/fmicb.2015.01362 (PMC4678201; doi:10.3389/fmicb.2015.01362)
Supplement: Supplementary file 1 [file Data_Sheet_1.DOCX]

**Supplementary material**

**Fig. S1. Diversity of bacteria detected in the tonsils (T) and mandibular lymph nodes (MLN). (A)** Rarefaction curves and **(B)** rank abundance curves based on an operational taxonomic unit (OTU) definition of 97% similarity (0.03 16S rRNA distance) are shown. Rarefaction and rank abundance curves were calculated for each sample. For better visualization, rank abundance curves were depicted as mean values per sampling site.


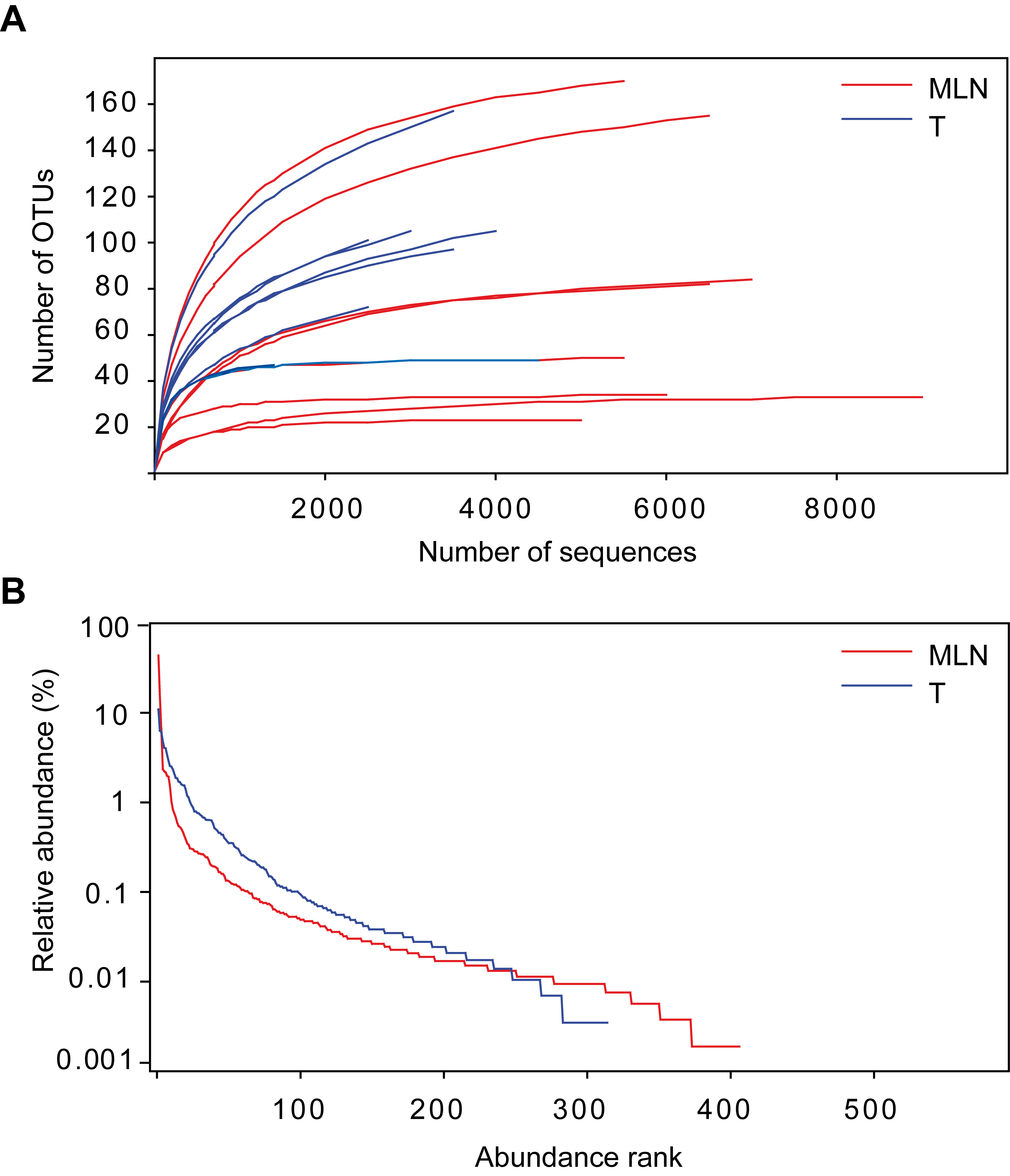


**Fig. S2. Venn diagram displaying the overlap between tonsil OTUs (Sanger Sequencing & cloning) published in Lowe and colleagues** (Lowe BA, Marsh TL, Isaacs-Cosgrove N, Kirkwood RN, Kiupel M, Mulks MH. 2011. Vet Microbiol 147:346-357) **and from the tonsil dataset (pyrosequencing) provided in this publication.** OTUs were classified based on 97% similarity.


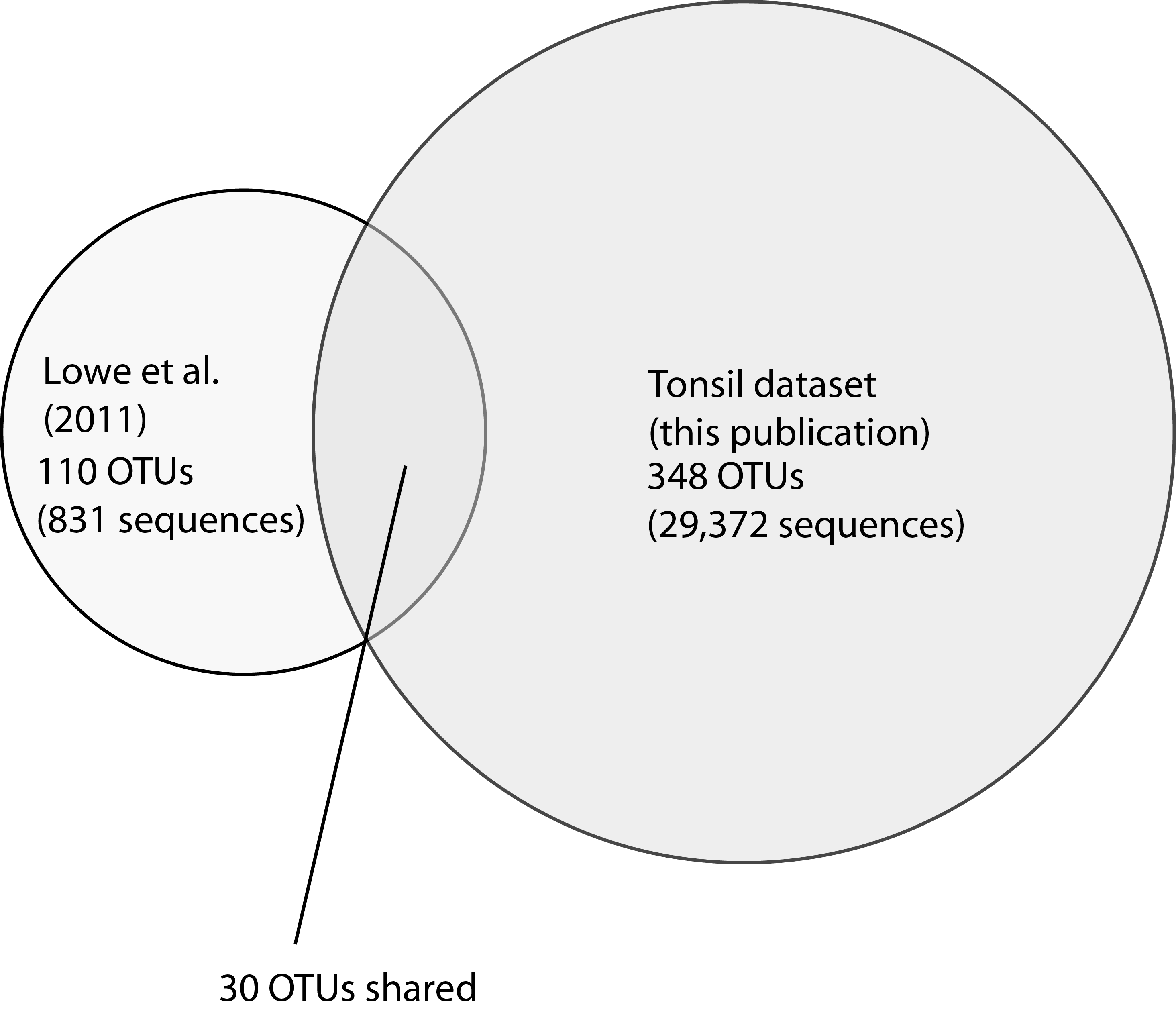


**Table S1.** Relative abundances of the most abundant OTUs of the RNA pyrosequencing dataset. For each group (T= tonsil, MLN= mandibular lymph node) the relative abundances of OTUs and standard deviation (SD) are listed. Groups were statistically compared and *p*-values are listed. The significance level was set to *p* < 0.05. To control the false discovery rate at 10%, only significant phylotypes with *q*-values ≤ 0.1 were considered (highlighted in orange).

|  |  |  |  |  |  |  |  |
| --- | --- | --- | --- | --- | --- | --- | --- |
|  |  |  |  |  |  |  |  |
| OTU No. | MLN | |  | T | |  | *p*-Values |
|  |  |  |  |  |  |  |  |
|  |  |  |  |  |  |  |  |
|  | Mean | SD |  | Mean | SD |  |  |
|  |  |  |  |  |  |  |  |
|  |  |  |  |  |  |  |  |
| OTU 1 | 41.8086 | 3.9725 |  | 5.2894 | 2.883 |  | 0.002997 |
| OTU 3 | 15.8656 | 3.0238 |  | 3.2186 | 2.5191 |  | 0.004995 |
| OTU 8 | 0.101 | 0.101 |  | 10.6438 | 3.3414 |  | 0.000999 |
| OTU 15 | 5.6005 | 1.0492 |  | 1.2251 | 0.9229 |  | 0.01 |
| OTU 18 | 0.6768 | 0.6768 |  | 6.4563 | 2.8572 |  | 0.092907 |
| OTU 24 | 0.0519 | 0.0463 |  | 4.595 | 1.8736 |  | 0.000999 |
| OTU 30 | 0.1664 | 0.1664 |  | 4.3713 | 2.9123 |  | 0.013986 |
| OTU 25 | - | - |  | 3.1495 | 2.4259 |  | - |
| OTU 6 | 2.1378 | 0.3655 |  | 0.7737 | 0.3167 |  | 0.018981 |
| OTU 31 | 2.2507 | 2.2507 |  | 0.5127 | 0.3208 |  | 0.004995 |
| OTU 52 | 0.0206 | 0.0206 |  | 3.6316 | 3.2471 |  | 0.095904 |
| OTU 46 | 0.1926 | 0.003 |  | 2.5489 | 1.1394 |  | 0.06993 |
| OTU 50 | 0.0224 | 0.0224 |  | 2.3387 | 1.1419 |  | 0.020979 |
| OTU 22 | 0.4618 | 0.4618 |  | 1.8454 | 0.599 |  | 0.098901 |
| OTU 12 | 0.0042 | 0.0042 |  | 2.0387 | 0.8788 |  | 0.000999 |
| OTU 40 | 0.1215 | 0.1215 |  | 2.0551 | 1.0548 |  | 0.020979 |
| OTU 42 | 2.3551 | 1.9226 |  | 0.0217 | 0.0157 |  | 0.125874 |
| OTU 45 | 1.8771 | 0.3217 |  | 0.07 | 0.0275 |  | 0.000999 |
| OTU 36 | 2.2295 | 1.8921 |  | 0.0797 | 0.0621 |  | 0.055944 |
| OTU 56 | 0.0206 | 0.0206 |  | 1.833 | 0.4992 |  | 0.000999 |
| OTU 54 | 0.1197 | 0.1197 |  | 1.9268 | 1.9268 |  | 0.460539 |
| OTU 43 | - | - |  | 1.6482 | 1.1344 |  | - |
| OTU 41 | 0.7245 | 0.3811 |  | 0.9229 | 0.4248 |  | 0.763237 |
| OTU 34 | 1.6673 | 0.9442 |  | 0.0806 | 0.0432 |  | 0.051948 |
| OTU 73 | - | - |  | 1.701 | 1 084 |  | - |
| OTU 7 | 0.002 | 0.002 |  | 1.474 | 0.022 |  | 0.138422 |
| OTU 86 | - | - |  | 1.252 | 0.103 |  | - |
| OTU 65 | 0.824 | 0.824 |  | 0.492 | 0.145 |  | 0.998002 |
| OTU 82 | 0.022 | 0.022 |  | 0.127 | 0.038 |  | 0.766696 |
| OTU 51 | 0.001 | 0.001 |  | 1 199 | 0.007 |  | 0.001998 |
|  |  |  |  |  |  |  |  |
|  |  |  |  |  |  |  |  |
|  |  |  |  |  |  |  |  |

**Table S2. Complete list of genera detected in the tonsils and mandibular lymph nodes (MLNs).** Genera are ranked by the decreasing number of read counts. Bootstrap values, indicating the confidence of the classification, were listed in brackets.

|  |  |
| --- | --- |
|  |  |
| **Genera-Tonsils** | **Genera-MLN** |
|  |  |
|  |  |
| *Prevotella(100)* | *Serratia(100)* |
| *Porphyromonas(100)* | *Herbaspirillum(100)* |
| *Campylobacter(100)* | *Pseudomonas(100)* |
| *Treponema(100)* | *Burkholderia(100)* |
| *Serratia(100)* | *Janthinobacterium(100)* |
| *Streptococcus(100)* | *Variovorax(100)* |
| *Herbaspirillum(100)* | *Propionibacterium(100)* |
| *Bacteroides(100)* | *Anoxybacillus(100)* |
| *Paraprevotella(98)* | *Corynebacterium(100)* |
| *Fusobacterium(100)* | *Methylobacterium(100)* |
| *Flavitalea(100)* | *Flavobacterium(100)* |
| *Alkanindiges(100)* | *Anaerovirgula(100)* |
| *Alysiella(100)* | *Streptococcus(100)* |
| *Dehalospirillum(100)* | *Hydrogenophilus(100)* |
| *Peptostreptococcus(100)* | *Treponema(100)* |
| *Pseudomonas(100)* | *Gemella(100)* |
| *Peptoniphilus(99)* | *Comamonas(100)* |
| *Sediminibacterium(86)* | *Staphylococcus(100)* |
| *Jonquetella(100)* | *Chryseobacterium(100)* |
| *Mycoplasma(100)* | *Lactobacillus(100)* |
| *Hydrogenophilus(100)* | *Porphyromonas(100)* |
| *Pasteurella(98)* | *Proteocatella(100)* |
| *Gemella(100)* | *Sphingomonas(100)* |
| *Chitinibacter(100)* | *Modestobacter(100)* |
| *Tannerella(96)* | *Acinetobacter(100)* |
| *Tessaracoccus(100)* | *Roseomonas(100)* |
| *Janthinobacterium(100)* | *Granulicatella(100)* |
| *Anaerovirgula(100)* | *Paracoccus(100)* |
| *Veillonella(100)* | *Bacteroides(100)* |
| *Thermovirga(54)* | *Fusobacterium(100)* |
| *Caloramator(100)* | *Stenotrophomonas(96)* |
| *Curvibacter(100)* | *Luteimonas(100)* |
| *Acetitomaculum(100)* | *Acidovorax(100)* |
| *Neisseria(100)* | *Prevotella(100)* |
| *Riemerella(97)* | *Paraprevotella(98)* |
| *Odoribacter(100)* | *Hymenobacter(100)* |
| *Peptostreptococcaceae(100)* | *Campylobacter(100)* |
| *Pelistega(100)* | *Amaricoccus(100)* |
| *Acidisoma(100)* | *Flavitalea(100)* |
| *Paludibacter(100)* | *Delftia(100)* |
| *Sebaldella(100)* | *Ferruginibacter(100)* |
| *Desulfovibrio(100)* | *Caloramator(100)* |
| *Cellvibrio(100)* | *Sulfurovum(88)* |
| *Propionivibrio(100)* | *Exiguobacterium(100)* |
| *Bergeyella(100)* | *Riemerella(97)* |
| *Anaerovibrio(80)* | *Nocardioides(100)* |
| *Selenomonas(100)* | *Lysobacter(95)* |
| *Actinobacillus(63)* | *Sebaldella(100)* |
| *Pseudoalteromonas(100)* | *Dehalospirillum(100)* |
| *Eubacterium(100)* | *Alkanindiges(100)* |
| *Anaerobiospirillum(100)* | *Cloacibacterium(100)* |
| *Staphylococcus(100)* | *Bacillus(100)* |
| *Anaerovorax(100)* | *Butyrivibrio(99)* |
| *Murdochiella(100)* | *Rhodococcus(100)* |
| *Stenotrophomonas(96)* | *Microbacterium(100)* |
| *Sediminicola(100)* | *Geobacillus(100)* |
| *Hallella(96)* | *Anaeroarcus(97)* |
| *Moraxella(100)* | *Simplicispira(90)* |
| *Propionibacterium(100)* | *Daeguia(100)* |
| *Atopobium(100)* | *Catenulispora(100)* |
| *Anoxybacillus(100)* | *Pelistega(100)* |
| *Bacillus(100)* | *Pelomonas(100)* |
| *Acinetobacter(100)* | *Leptotrichia(100)* |
| *Variovorax(100)* | *Pseudoxanthomonas(100)* |
| *Proteocatella(100)* | *Soonwooa(100)* |
| *Tepidimicrobium(100)* | *Fervidobacterium(100)* |
| *Centipeda(100)* | *Clostridium_XI(100)* |
| *Mogibacterium(96)* | *Solirubrobacter(100)* |
| *Dialister(100)* | *Aeribacillus(100)* |
| *Lactobacillus(100)* | *Aminobacterium(97)* |
| *Negativicoccus(100)* | *Pedobacter(100)* |
| *Filifactor(100)* | *Sphingobacterium(95)* |
| *Methylobacterium(100)* | *Brevundimonas(100)* |
| *Actinomyces(100)* | *Petrobacter(100)* |
| *Lacibacter(84)* | *Schlegelella(100)* |
| *Sharpea(100)* | *Aciditerrimonas(100)* |
| *Kineococcus(100)* | *Pasteurella(98)* |
| *Sporotalea(100)* | *Thermus(100)* |
| *Halomonas(100)* | *Hellea(100)* |
| *Corynebacterium(100)* | *Sediminibacterium(86)* |
| *Clostridium_sensu_stricto(100)* | *Veillonella(100)* |
| *Alistipes(67)* | *Novosphingobium(100)* |
| *Abiotrophia* | *Actinotalea(100)* |
| *Kingella(100)* | *Micrococcus(100)* |
| *Catonella(100)* | *Marvinbryantia(100)* |
| *Megasphaera(100)* | *Lachnospiracea_incertae_sedis(67)* |
| *Elizabethkingia(100)* | *Dietzia(100)* |
| *Marvinbryantia(100)* | *Oscillibacter(100)* |
| *Parvimonas(100)* | *Ramlibacter(100)* |
| *Haemophilus(75)* | *Kocuria(100)* |
| *Exilispira(89)* | *Clostridium_sensu_stricto(100)* |
| *Burkholderia(100)* | *Leucobacter(100)* |
| *Fluviicola(100)* | *Ralstonia(100)* |
| *Castellaniella(100)* | *Truepera(100)* |
| *Kiloniella(89)* | *Rothia(100)* |
| *Parabacteroides(100)* | *Pseudoclavibacter(100)* |
| *Proteiniborus(100)* | *Blastococcus(100)* |
| *Butyrivibrio(99)* | *Deinococcus(100)* |
| *Petrobacter(100)* | *Giesbergeria(100)* |
| *Clostridium_XI(100)* | *Chitinibacter(100)* |
| *Comamonas(100)* | *Blastomonas(100)* |
| *Anaerorhabdus(100)* | *Beijerinckia(100)* |
| *Tepidimonas(100)* | *Empedobacter(100)* |
| *Acidaminococcus(100)* | *Microvirga(100)* |
| *Capnocytophaga(88)* | *Wenxinia(100)* |
| *Fibrobacter(100)* | *Acidisoma(100)* |
| *Fervidobacterium(100)* | *Neisseria(100)* |
| *Rothia(100)* | *Atopobium(100)* |
| *Guggenheimella(100)* | *Succinivibrio(100)* |
| *Thermonema(100)* | *Butyricicoccus(100)* |
| *Conchiformibius(100)* | *Anaerovibrio(80)* |
| *Dokdonia(100)* | *Mitsuokella(100)* |
| *Streptobacillus(100)* | *Haloplasma(100)* |
| *Dorea(97)* | *Peptostreptococcus(100)* |
| *Cetobacterium(98)* | *Alysiella(100)* |
| *Bilophila(100)* | *Hallella(96)* |
| *Azonexus(86)* | *Cellvibrio(100)* |
| *Thermus(100)* | *Turicibacter(100)* |
| *Proteus(100)* | *Actinomyces(100)* |
| *Pelomonas(100)* | *Elizabethkingia(100)* |
| *Joostella(90)* | *Bradyrhizobium(100)* |
| *Phyllobacterium(100)* | *Anaerostipes(100)* |
| *Thermodesulfobacterium(100)* | *Humicoccus(100)* |
| *Suttonella(100)* | *Tannerella(96)* |
| *Helicobacter(100)* | *Vagococcus(100)* |
| *Acidovorax(100)* | *Byssovorax(100)* |
| *Turicibacter(100)* | *Finegoldia(100)* |
| *Delftia(100)* | *Mucilaginibacter(100)* |
| *Daeguia(100)* | *Helicobacter(100)* |
| *Oceanotoga(97)* | *Alloiococcus(99)* |
| *Schlegelella(100)* | *Sphingobium(100)* |
| *Fusibacter(100)* | *Curvibacter(100)* |
| *Succinivibrio(100)* | *Phyllobacterium(100)* |
|  | *Thiofaba(100)* |
|  | *Thermodesulfobacterium(100)* |
|  | *Atopostipes(100)* |
|  | *Cupriavidus(100)* |
|  | *Duganella(58)* |
|  | *Goodfellowiella(100)* |
|  | *Micromonospora(100)* |
|  | *Odoribacter(100)* |
|  | *Phocaeicola(100)* |
|  | *Selenomonas(100)* |
|  | *Sutterella(100)* |
|  | *Cesiribacter(100)* |
|  | *Paucisalibacillus(100)* |
|  | *Pseudoalteromonas(100)* |
|  | *Brevibacterium(100)* |
|  | *Roseburia(61)* |
|  | *Anaerococcus(100)* |
|  | *Bosea(100)* |
|  | *Cellulosilyticum(100)* |
|  | *Escherichia_Shigella(100)* |
|  | *Abiotrophia* |
|  | *Mycoplasma(100)* |
|  | *Tepidimicrobium(100)* |
|  | *Dorea(97)* |
|  | *Negativicoccus(100)* |
|  | *Mogibacterium(96)* |
|  | *Rubrivivax(100)* |
|  | *Faecalibacterium(100)* |
|  | *Achromobacter(86)* |
|  | *Anaerophaga(100)* |
|  | *Xylanibacter(100)* |
|  | *Zhouia(80)* |
|  | *Macrococcus(100)* |
|  | *Sediminicola(100)* |
|  | *Anaerobacter(100)* |
|  | *Suttonella(100)* |
|  | *Anaerorhabdus(100)* |
|  | *Cetobacterium(98)* |
|  | *Lactonifactor(89)* |
|  | *Filifactor(100)* |
|  | *Citrobacter(100)* |
|  | *Psychrobacter(100)* |
|  | *Brochothrix(100)* |
|  | *Paludibacter(100)* |
|  | *Peptostreptococcaceae(100)* |
|  | *Enhydrobacter(100)* |
|  | *Rikenella(100)* |
|  | *Halomonas(100)* |
|  | *Lacibacter(84)* |
|  | *Paenibacillus(100)* |
|  | *Janibacter(100)* |
|  |  |
|  |  |
|  |  |
|  |  |
|  |  |
|  |  |
|  |  |
|  |  |
|  |  |
|  |  |
|  |  |
|  |  |
|  |  |
|  |  |
|  |  |
|  |  |
|  |  |
|  |  |
|  |  |
|  |  |
|  |  |
|  |  |
|  |  |
|  |  |
|  |  |
|  |  |
|  |  |
|  |  |

**Table S3 Relative abundances of the most abundant genera over both sampling sites.** For each group (T= tonsil-, MLN=mandibular lymph nodes) the relative abundances of genera and standard deviation (SD) are listed. Groups were statistically compared and *p*-values are listed. The significance level was set to *p* < 0.05. To control the false discovery rate at 10%, only significant phylotypes with *q*-values ≤ 0.1 were considered (highlighted in orange).

|  |  |  |  |  |  |  |  |  |
| --- | --- | --- | --- | --- | --- | --- | --- | --- |
|  |  |  |  |  |  |  |  |  |
|  |  | MLN |  |  | T |  |  | *p*-values |
|  |  |  |  |  |  |  |  |  |
| Genus |  |  |  |  |  |  |  |  |
|  |  | Mean | SD |  | Mean | SD |  |  |
|  |  |  |  |  |  |  |  |  |
|  |  |  |  |  |  |  |  |  |
| *Serratia* |  | 48.6512 | 6.156 |  | 6.5675 | 3.4905 |  | 0.000999 |
| *Herbaspirillum* |  | 19.0525 | 3.403 |  | 3.8922 | 2.9853 |  | 0.003996 |
| *Pseudomonas* |  | 6.6632 | 1.0067 |  | 1.4981 | 1.0945 |  | 0.006993 |
| *Prevotella* |  | 0.3567 | 0.1786 |  | 17.6468 | 5.2655 |  | 0.001998 |
| *Porphyromonas* |  | 0.7411 | 0.6532 |  | 12.5502 | 3.358 |  | 0.000999 |
| *Treponema* |  | 0.9297 | 0.9297 |  | 11.4852 | 4.2528 |  | 0.017982 |
| *Campylobacter* |  | 0.2532 | 0.1443 |  | 12.118 | 4.4018 |  | 0.000999 |
| *Streptococcus* |  | 1.288 | 0.6624 |  | 8.0419 | 3.8658 |  | 0.01998 |
| *Fusobacterium* |  | 0.3761 | 0.3669 |  | 3.924 | 0.9959 |  | 0.005994 |
| *Bacteroides* |  | 0.4287 | 0.2799 |  | 4.7923 | 1.571 |  | 0.021978 |
| *Acidisoma* |  | 0.0319 | 0.0319 |  | 0.3293 | 0.3195 |  | 0.450549 |
| *Helicobacter* |  | 0.0168 | 0.0138 |  | 0.0043 | 0.0043 |  | 0.474525 |
| *Paraprevotella* |  | 0.3514 | 0.2024 |  | 4.967 | 1.348 |  | 0.002997 |
| *Janthinobacterium* |  | 2.6622 | 0.3298 |  | 0.6307 | 0.386 |  | 0.001998 |
| *Anoxybacillus* |  | 2.134 | 1.1985 |  | 0.1071 | 0.0568 |  | 0.02997 |
| *Propionibacterium* |  | 3.0346 | 2.5498 |  | 0.0965 | 0.0736 |  | 0.041958 |
| *Burkholderia* |  | 4.6285 | 3.6901 |  | 0.0349 | 0.0222 |  | 0.011988 |
| *Lactobacillus* |  | 1.0223 | 0.8374 |  | 0.0773 | 0.0642 |  | 0.198801 |
| *Acinetobacter* |  | 0.442 | 0.345 |  | 0.0754 | 0.0624 |  | 0.322677 |
| *Hydrogenophilus* |  | 0.9503 | 0.4732 |  | 1.2217 | 0.5573 |  | 0.751249 |
| *Variovorax* |  | 2.2239 | 0.3188 |  | 0.0892 | 0.0349 |  | 0.000999 |
| *Corynebacterium* |  | 1.8766 | 0.8781 |  | 0.0508 | 0.0301 |  | 0.002997 |
| *Caloramator* |  | 0.224 | 0.1195 |  | 0.4629 | 0.2232 |  | 0.42957 |
| *Alloiococcus* |  | 0.0229 | 0.0156 |  | - | - |  | - |
| *Dehalospirillum* |  | 0.1742 | 0.1742 |  | 2.7382 | 2.7382 |  | 0.471528 |
| *Flavitalea* |  | 0.2526 | 0.2526 |  | 3.2979 | 1.4785 |  | 0.061938 |
| *Alysiella* |  | 0 | 0 |  | 2.5542 | 1.5945 |  | 0.004 |
| *Anaerovirgula* |  | 1.1848 | 1.1848 |  | 0.7464 | 0.2163 |  | 0.99 |
|  |  |  |  |  |  |  |  |  |
|  |  |  |  |  |  |  |  |  |

**Table S4 Relative abundances of phyla detected.** For each group (T= tonsil-, MLN= mandibular lymph node) the relative abundances of phyla and standard deviation (SD) are listed. Groups were statistically compared and *p*-values are listed. The significance level was set to *p* < 0.05. To control the false discovery rate at 10%, only significant phylotypes with *q*-values ≤ 0.1 were considered (highlighted in orange).

|  |  |  |  |  |  |  |  |  |
| --- | --- | --- | --- | --- | --- | --- | --- | --- |
|  |  |  |  |  |  |  |  |  |
| Phylum |  | MLN | |  | T | |  | *p*-values |
|  |  |  |  |  |  |  |  |  |
|  |  |  |  |  |  |  |  |  |
|  |  | Mean | SD |  | Mean | SD |  |  |
|  |  |  |  |  |  |  |  |  |
|  |  |  |  |  |  |  |  |  |
| *Proteobacteria* |  | 81.374 | 4.256 |  | 33.087 | 7.968 |  | 0.002 |
| *Firmicutes* |  | 7.748 | 10.830 |  | 14.266 | 6.422 |  | 0.137 |
| *Bacteroidetes* |  | 4.477 | 15.321 |  | 36.577 | 3.978 |  | 0.002 |
| *Actinobacteria* |  | 4.600 | 18.214 |  | 0.960 | 20.738 |  | 0.171 |
| *Fusobacteria* |  | 0.513 | 28.236 |  | 3.599 | 10.629 |  | 0.006 |
| *Spirochaetes* |  | 0.743 | 32.787 |  | 8.147 | 11.912 |  | 0.017 |
| *Tenericutes* |  | 0.034 | 18.162 |  | 0.967 | 12.369 |  | 0.002 |
| *Synergistetes* |  | 0.062 | 31.942 |  | 1.457 | 29.407 |  | 0.184 |
| *SR1* |  | 0.150 | 33.072 |  | 0.395 | 14.335 |  | 0.346 |
| *Deinococcus-Thermus* |  | 0.105 | 16.439 |  | 0.010 | 16.137 |  | 0.062 |
| *TM7* |  | 0.002 | 33.072 |  | 0.432 | 32.775 |  | 0.261 |
| *Thermotogae* |  | 0.064 | 17.260 |  | 0.017 | 19.843 |  | 0.188 |
| *Acidobacteria* |  | 0.105 | 33.072 |  | 0.014 | 33.072 |  | 0.593 |
| *Fibrobacteres* |  | 0.011 | 33.072 |  | 0.048 | 33.072 |  | 0.470 |
| *Chloroflexi* |  | - | - |  | 0.017 | 17.139 |  | - |
| *Thermodesulfobacteria* |  | 0.013 | 28.065 |  | 0.007 | 33.072 |  | 0.506 |
|  |  |  |  |  |  |  |  |  |
|  |  |  |  |  |  |  |  |  |
